# Nanoparticle vaccines based on the receptor binding domain of porcine deltacoronavirus elicit robust protective immune responses in mice

**DOI:** 10.3389/fimmu.2024.1328266

**Published:** 2024-03-14

**Authors:** Yuanhong Wang, Junhan Song, Xiaoying Deng, Junna Wang, Miao Zhang, Yun Liu, Pan Tang, Huili Liu, Yanjun Zhou, Guangzhi Tong, Guoxin Li, Lingxue Yu

**Affiliations:** ^1^ Shanghai Veterinary Research Institute, Chinese Academy of Agricultural Sciences, Shanghai, China; ^2^ Institute of Animal Husbandry and Veterinary Science, Shanghai Academy of Agricultural Sciences, Shanghai, China; ^3^ Jiangsu Co-innovation Center for Prevention and Control of Important Animal Infectious Diseases and Zoonoses, Yangzhou University, Yangzhou, China

**Keywords:** porcine deltacoronavirus, nanoparticle vaccine, ferritin, SpyTag/SpyCatcher, RBD

## Abstract

**Background:**

Porcine deltacoronavirus (PDCoV), a novel swine enteropathogenic coronavirus, challenges the global swine industry. Currently, there are no approaches preventing swine from PDCoV infection.

**Methods:**

A new PDCoV strain named JS2211 was isolated. Next, the dimer receptor binding domain of PDCoV spike protein (RBD-dimer) was expressed using the prokaryotic expression system, and a novel nanoparticle containing RBD-dimer and ferritin (SC-Fe) was constructed using the SpyTag/SpyCatcher system. Finally, the immunoprotection of RBD-Fe nanoparticles was evaluated in mice.

**Results:**

The novel PDCoV strain was located in the clade of the late Chinese isolate strains and close to the United States strains. The RBD-Fe nanoparticles were successfully established. Immune responses of the homologous prime-boost regime showed that RBD-Fe nanoparticles efficiently elicited specific humoral and cellular immune responses in mice. Notably, high level PDCoV RBD-specific IgG and neutralizing antibody (NA) could be detected, and the histopathological results showed that PDCoV infection was dramatically reduced in mice immunized with RBD-Fe nanoparticles.

**Conclusion:**

This study effectively developed a candidate nanoparticle with receptor binding domain of PDCoV spike protein that offers protection against PDCoV infection in mice.

## 1 Introduction

Porcine deltacoronavirus (PDCoV) is a novel enteropathogenic coronavirus. It leads to any age of pigs especially newborn piglets developed gastrointestinal symptoms such as diarrhea and vomiting. Newborn piglets are highly susceptible to PDCoV infection, resulting in mortality rates ranging from 30% to 40% (1). Since PDCoV was first reported in 2012, the newly emerged PDCoV have spread worldwide, causing a high number of pig deaths and significant economic impacts (1–7).

PDCoV has a broad host range, including mammals and avains (8, 9). Moreover, a case report in 2021 has identified PDCoV in plasma samples of three Haitian children with acute undifferentiated febrile illness (10). The rapid transmission and potential for interspecies transmission of PDCoV pose significant threats to both human and animal health. Nonetheless, there are presently no commercially available vaccines for the prevention and control of PDCoV. This underscores the pressing need for PDCoV vaccine development (11).

Among various vaccine platforms, subunit vaccines typically offer excellent safety profiles, rapid production, and ease of scalability. PDCoV enters the cell via the RBD region of spike protein binding the aminopeptidase N (APN) (12, 13). Currently, comprehensive understanding of the structures and biological function of the PDCoV spike protein has motivated the RBD as the vaccine immunogen. And the recombinant RBD proteins derived from other coronaviruses such as SARS-CoV, SARS-CoV-2, and MERS-CoV have previously demonstrated their immunogenicity, effectively eliciting protective neutralizing antibodies in animal models (14, 15).

However, the application of RBD-based subunit vaccines as candidate vaccines is hindered by low immunogenicity (16). To enhance immunogenicity multimerization modified strategy has been used for vaccine development. And the modified vaccines have induced significantly immune responses to target pathogens (17, 18). In recent years, there has been notable advancement in protein covalent linkage strategies, simplifying protein modification and multimerization. Since the inception of the bacterially derived self-assembling SpyTag/SpyCatcher system in 2012, this linkage system has undergone refinement to enhance its efficiency and stability (19). This approach allows for rapid protein purification and macromolecular assembly, making it suitable for vaccine development (20). Previous works with HBV and HIV vaccines using SpyTag/SpyCatcher to present antigens on nanoparticle scaffolds has shown improved immunogenicity compared to unattached monomers (21, 22). In addition, ferritin nanoparticles, composed of 24 copies of the same ferritin subunits, self-assemble to form a highly symmetrical octahedral cage-like structure, showing significant thermal and chemical stability, making them suitable carriers for drug delivery and scaffolds for displaying exogenous peptides or protein (23–25). In this study, we constructed RBD-Fe nanoparticles by covalentially coupling PDCoV RBD-dimer and SC-Fe using the SpyTag/SpyCatcher system and evaluated the immunoprotection in mice. These data showed that we have developed a low-cost and effective candidate vaccine against PDCoV.

## 2 Materials and methods

### 2.1 Cells, and animals

LLC-PK1 cells (porcine kidney cells) were maintained in our laboratory, and grow in Dulbecco’s modified Eagle’s medium (DMEM) (Hyclone, USA) supplemented with trypsin (Gibico, Australia) in 5% CO_2_ at 37°C. Animal experiments were conducted following the guidelines approved by the Experimental Animal Care and Use Committee of the Shanghai Veterinary Research Institute, Chinese Academy of Agricultural Sciences (No. SV-20230512-02).

### 2.2 Sample collection

Fecal samples were collected from piglets with diarrhea on a piggery in Jiangsu, China. RT-PCR was performed to identify the PDCoV and excluded the Porcine epidemic diarrhea virus (PEDV), TGEV and porcine rotavirus (PRoV) infection using cDNA templates synthesized from the RNA extracted from fecal samples and specific primer pairs (**Table 1**).

**Table 1:** Specific primer pairs to identify PDCoV.

Primers	Sequences (5’-3’)	Annealing temperature (°C)	GenBank ID	Amplification size
PDCoV	CTTAAGTATGGTGAACTCCCTCCTAATG	60	MN942260.2	245 bp
PDCoV	GATTGAGATCTTGGGCCACTTCCACGC			
PEDV-F	GTAATTCACAGAATCTTGGAAATAAC	58	OP784565.1	241 bp
PEDV-R	GACCTTTCCTGTTTGGGCTTCTGCTG			
TGEV-F	GACACAGAAAAACAACAGCAACGCTC	58	DQ811785.1	486 bp
TGEV-R	GTAATTTTCTATTAATGCATCAGGTAC			
PRoV-F	GATTATTCATGCGCTTTAAATGCACC	56	KT820772.1	462 bp
PRoV-R	CGTTACATTTGCCAATAAAGTTTCTG			

### 2.3 Isolation of PDCoV JS2211 strain

To isolate PDCoV, the fecal sample was aseptically treated and inoculated it into a monolayer LLC-PK1 cells with trypsin. When approximately 80-90% of the cells exhibited cytopathic effect (CPE), repeated freezing and thawing was performed, and then cell debris was removed by centrifugation at 1500×g for 5 minutes at room temperature. Viral titer was measured using 50% tissue culture infectious dose (TCID_50_) assays on LLC-PK1 cells in a 96‐well plate.

### 2.4 Immunofluorescence assays and Western blot

LLC-PK1 cells in 6-well plates were infected with the PDCoV at a 0.1 multiplicity of infection (MOI) and fixed with 4% paraformaldehyde after 24 hpi. Subsequently, PDCoV N-protein polyclonal antibody which were produced from PDCoV N-immunized BALB/c mice was used as the primary antibody (1:100), followed by fluorescent isothiocyanate (FITC)-labeled goat anti-mouse immunoglobulin-G (IgG) secondary antibody (1:10000) (Invitrogen, 31569), after counterstained with 4’,6-diamidino-2-phenylindole (DAPI) at room temperature for 5min, the fluorescence were detected by microscope (Nikon,Japan). Mock infected LLC-PK1 cells served as a negative control.

In addition, when >80% CPE was evident in the inoculated cell monolayers (around post inoculate day 2), the plates were frozen at −80°C and thawed twice. the cell lysates were prepared for 12% sodium dodecyl sulfate-polyacrylamide gel electrophoresis (SDS-PAGE), and proteins were electroblotted onto a polyvinylidene difluoride membrane (Bio-Rad, USA). The primary antibody PDCoV N-protein polyclonal antibody and the secondary antibody horseradish peroxidase (HRP) conjugated goat anti-mouse IgG (ZSBio, ZB2305) (1:5000) were subjected to the Western blot analysis.

### 2.5 Phylogenetic analysis

The complete genome of novel isolated PDCoV strain named PDCoV JS2211 was obtained by using next-generation sequencing (tpbio Co., LTd). For comparing and sorting the spike gene, ModelFinder software was used to select best model which is TN+F+93. The Maximum Likelihood (ML) tree obtained after 10,000 calculations, and the final ML tree was optimized using the iTOL website.

### 2.6 Plasmid construction and protein expression

6x His-tagged *Helicobacter pylori* nonheme iron-containing ferritin (GenBank accession no. NP223316) and PDCoV spike RBD-dimer (GenBank accession no. MW349841.1) were synthesized by Sangon Biotech. Additionally, Spy Tag (ST) (13 amino acids) was fused to the N-terminus of RBD-dimer (ST-RBD), and Spy Catcher (SC) (113 amino acids) was fused to the N-terminus of ferritin (SC-Fe). SC-Fe and ST-RBD were separately cloned into the pET32a vector and sequenced by Sangon Biological Co., LTD. The verified vectors were transformed into BL21 competent cells (Takara) to introduce the protein expression. Single clone was amplified in LB with ampicillin at 37°C to an OD600 of 0.4-0.6. Bacteria solution was added with 0.1mM/L isopropyl β-D-1thiogalactopyranoside (IPTG) to induce protein expression. After 4h post induction, protein-expressing bacteria were harvested by centrifugation and pellets washed twice were suspended in sterile PBS. After lysed by sonication, the supernatants were incubated with Ni-NTA agarose to enrich His-tagged SC-Fe and ST-RBD. Following purification, the concentrated proteins were quantified using the BCA assay (26).

### 2.7 Protein extracellular self-assembly *in vitro*

ST-RBD and SC-Fe were mixed in a 1:1 molar ratio at 4°C overnight to produce the assembly RBD-Fe nanoparticle. Western blotting was performed to verify the self-assembly of the RBD-Fe using mouse anti-His-Tag antibody. Furthermore, the endotoxin levels in SC-Fe, ST-RBD, and assembled protein RBD-Fe were assessed using tachypleus amebocyte lysate test (less than 10 EU/dose).

### 2.8 Transmission electron microscopy

5 μL of SC-Fe and RBD-Fe nanoparticles were stained with 2% phosphotungstic acid and imaged using a Tecnai G2 Spirit BIOTWIN electron microscope (ThermoFisher) operated at an accelerating voltage of 80 kV.

### 2.9 Immunofluorescence identification

5 μL of SC-Fe and RBD-Fe nanoparticles were subjected to centrifugation to collect the precipitate. The precipitate was then washed with PBS, followed by fixation and blocking with 5% BSA-PBS. After centrifugation and removing the supernatant, 100 μL of mouse anti-PDCoV RBD polyclonal antibody prepared by our laboratory (1:400) and 100 μL of rabbit anti-ferritin monoclonal antibody (Abcam, ab75973) (1:1000) were added and incubated overnight at 4°C. After three times washing with PBS, 100 μL of Alexa Fluor 594 goat anti-rabbit IgG (Invitrogen, A-11012)(1:500) and Alexa Fluor 488 goat anti-mouse IgG (Invitrogen, A-10680) (1:500) were added and incubated at 37°C for 1 hour protected from light. After three times washing with PBS, the samples were resuspended in 100 μL of PBS. The particles were transferred to slides, coverslipped and sealed with neutral resin. Finally, the fluorescence was confirmed by Zeiss fluorescence microscope.

### 2.10 Mouse challenge and protection efficacy evaluation

Six-week-old Kunming mice (n=40) were randomly divided into four groups (n=10; SC-Fe, RBD-Fe, challenge, and control groups). Antigens were mixed with ISA 201VG adjuvant (Seppic, France) in a 50:50 w/w ratio. In the SC-Fe and RBD-Fe group, mice received subcutaneous injection of 100 μg mixtures, respectively. The Challenge and Control groups received an equivalent volume of PBS. A booster immunization was administered two weeks later. Blood samples were collected on 0, 2, 4 and 6 weeks post-immunization through retro-orbital bleeding. Serum was isolated and heat-inactivated at 56°C for 30 minutes and stored at -80°C.

At week 4, mice in the SC-Fe, RBD-Fe, and challenge groups were orally administered 200 μL and intramuscularly injected with 100 μL of PDCoV strain JS2211 (TCID_50 =_ 10^7.6^/mL), respectively (27). Control group mice were received an equivalent volume of physiological saline via the same route. Then, mice were observed daily for clinical symptoms. Mice were euthanized at week 6, and tissues (lungs, stomach, small intestine) were collected for PDCoV detection and histopathological observation.

### 2.11 Serological analysis

PDCoV-RBD specific IgG was evaluated from the serum collected on 0, 2, 4 and 6 weeks post-immunization by ELISA assay. Briefly, Recombinant ST-RBD was coated on high-binding 96-well plates at 100 ng per well and incubated overnight at 4°C. After washing three times with PBST, plates were blocked with 5% skim milk at 37°C for 2 hours. Following another three washes with PBST, immunized animal serum were serially diluted and added into each well in duplicate followed by incubating at 37°C for 1 hour. After three washes with PBST, HRP-conjugated goat anti-mouse IgG (1:5000 diluted in PBST) was added and incubated at 37°C for 1 hour. The reaction was terminated with 2M‐H_2_SO_4_ after incubation with 3,3′,5,5′-tetramethylbenzidine (TMB) for 15 minutes at room temperature. The OD value was measured at 450 nm using a microplate reader.

### 2.12 Neutralizing antibody detection

Virus neutralizing antibody test was performed using PDCoV JS2211 to determine the neutralizing antibodies (NA) in mouse serum collected at week 4. 100 μL of serum at two-fold serial dilutions were mixed with 100 μL of DMEM containing 200 TCID_50_ PDCoV with trypsin and incubated at 37°C for 1 hour to inoculate LLC-PK1 cells. The highest dilution of serum that showed at least 50% CPE compared to the negative control was determined as the NA titer.

### 2.13 PDCoV detection

Approximately 1g of lung, stomach, colon, and duodenum tissues collected from mice in each group were homogenized using a 3D cryogenic grinder (servicebio), respectively. RNA was extracted using a DNA/RNA Extraction Kit FT (vazyme). Subsequently, cDNA was synthesized from 2 µg of total RNA using 5× PrimeScript RT Master Mix (Takara, RR036Q). Primers N-F (5′- TGCTACCTCTCCGATTCCCAACC -3′), N-R (5′- GCTGATTGCCTGTGCCTCTGG −3′), β-actin-F (5′- TATGCTCTCCCTCACGCCATCC -3′), and β-actin-R (5′- GTCACGCACGATTTCCCTCTCAG −3′) were designed and synthesized for qRT-PCR to determine the relative levels of mRNA in different tissues. The reaction conditions were as follows: 95°C initial denaturation for 60 s; 94°C denaturation for 5 s, 60°C annealing for 30 s; 40 cycles of amplification; melting curve analysis at 95°C for 10 s, 65°C for 60 s, and 97°C for 1 s. Viral infection in various organ tissues was analyzed using the 2^-ΔΔCt^ method.

### 2.14 Histopathological analysis

The duodenum from each group of mice was fixed in 4% paraformaldehyde for 48 hours and subsequently embedded in paraffin. Sections (3-4 µM) were stained with hematoxylin and eosin (H&E). For immunohistochemistry, sections were incubated overnight at 4°C with rabbit anti-PDCoV N polyclonal antibody at a dilution of 1:200. Subsequently, sections were incubated with goat anti-rabbit IgG secondary antibody (HRP) for 2 hours at room temperature, followed by staining with 3,3’-diaminobenzidine (DAB). For IFA staining, sections were incubated overnight at 4°C with rabbit anti-PDCoV N polyclonal antibody at a dilution of 1:200. Subsequently, sections were incubated with Alexa Fluor^®^ 680-conjugated donkey anti-rabbit IgG (Servicebio) for 60 minutes and counterstained with DAPI for nuclear staining. Microscopic observations and photography were performed.

### 2.15 Flow cytometry analysis

At week 6, spleens from each group were collected in RPMI medium containing 2% FBS (Hyclone) and single-cell suspensions were prepared as previously described (28). For staining of T and B cell surface markers, live cells were stained with fluorescently conjugated monoclonal antibodies in PBS containing 0.5% BSA at 4°C for 30 min protected from light. The following antibodies were used: anti-CD3 (Invitrogen, 12-0031-82), anti-CD4 (Invitrogen, 11-0041-82), anti-CD8 (Invitrogen, 25-0081-82), and anti-CD19 (Invitrogen, 48-0193-82). Data acquisition and analysis were performed using an ACEN flow cytometer.

### 2.16 Statistical analysis

Values are shown as the mean ± standard error (s.e.m.) and the collected experimental data were analyzed using GraphPad Prism 7.0. Independent sample t-tests were used for comparisons between two groups, and one-way ANOVA followed by *post hoc* tests were used for comparisons among multiple groups. *P* value of less than 0.05 was considered statistically significant. Homology modeling of PDCoV’s RBD-dimer and ferritin structures was performed using Alphafold2, and model images generated by Alphafold2 were further optimized using PyMOL (29).

## 3 Results

### 3.1 Isolation and biological characteristics of the PDCoV JS2211 strain

**Figure 1:**
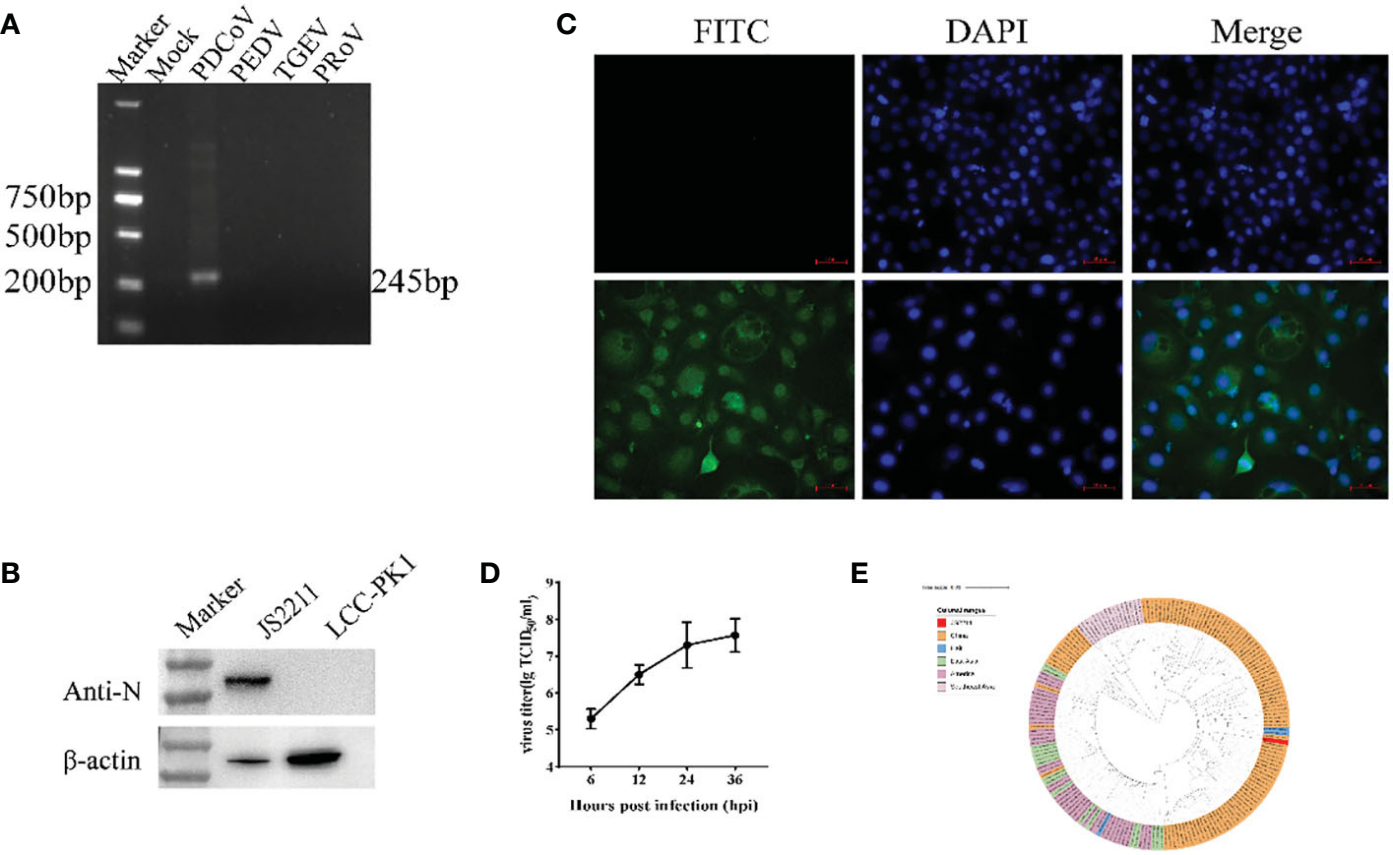
Characteristics of isolated PDCoV. **(A)** RT-PCR assay to identify PDCoV infection. **(B)** Immunofluorescence assay of JS2211 in LLC-PK1 cells. **(C)** Western blot analysis of JS2211 proliferation in LLC-PK1 cells. N protein was detected by anti-PDCoV N polyclonal antibody (top) and β-actin served as loading control (bottom); **(D)** Multistep replication curves of JS2211 in LLC-PK1 cells at 6, 12, 24 and 36 hpi. **(E)** The ML tree of PDCoV was deduced using the Spike gene of the different PDCoV. The different coronaviruses are expressed by different RGB colors as indicated. JS2211 is labeled in red.

The RT-PCR results showed that the sample were positive for PDCoV with the negative-PEDV,TGEV and PRoV (**Figure 1A**). The feacal sample was passed through 0.22-μm filters and used to inoculate LLC-PK1 cells. After 24 h, LLC-PK1 cells exhibited CPE, including shrinking, rounding, lighting, and disruptive morphological characteristics, and the cells were subjected to Western blot and IFA staining. As shown in **Figure 1B**, PDCoV JS2211 was used to infect LLC-PK1 cells, and N-specific fluorescence was observed by IFA (**Figure 1C**). Moreover, multistep replication curves revealed that mean virus titer of JS2211 was reached 10^7.6^ TCID_50_ at 36 hpi (**Figure 1D**).

The results of the ML tree showed that the JS2211 strains located in the clade of the late Chinese isolate strains and close to the United States strains, indicating that JS2211 is a relatively new strain, and probably came from the United States through international trade and became popular in China (**Figure 1E**). Furthermore, the JS2211 was closed with the Haiti strains which could infect child. The spread of PDCoV is relatively fast, suggests that development a PDCoV vaccines is necessary for both the livestock industry and human health.

### 3.2 Characterization of the recombinant protein

**Figure 2:**
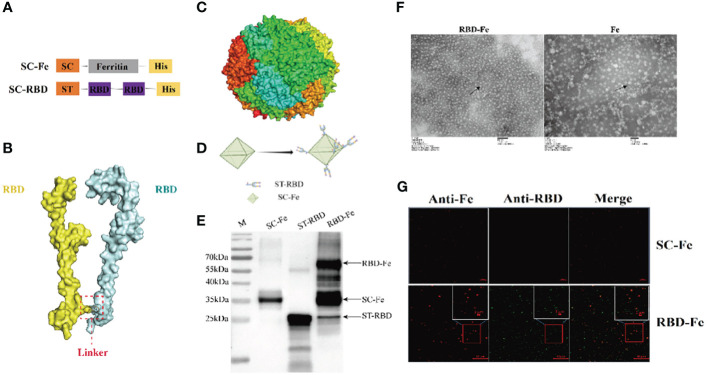
Construction and identification of RBD-Fe Nanoparticle Vaccine. **(A)** Schematic of vaccine components which were 6 × His-tagged SC-Ferritin and ST-RBD. SC: SpyCatcher. ST: SpyTag. **(B)** A schematic diagram of the structure simulation of the RBD-dimer using AlphaFold2. **(C)** Schematic diagram of the structure simulation of the Fe protein using AlphaFold2. **(D)** Schematic illustration of RBD-Fe nanoparticles. **(E)** Western blot analysis of the ST-RBD, SC-Fe and RBD-Fe. **(F)** Negative-staining EMs of unlinked nanoparticles SC-Fe and RBD-Fe NPs. **(G)** Confocal microscopy imaging of Fe and RBD-Fe nanoparticles.

The SpyTag (ST) (13 aa) was genetically fused at the N terminus of RBD and SpyCatcher (SC) (113 aa) was genetically fused at the N-terminus of ferritin at the downstream of 6xHis-tag (**Figure 2A**). AlphaFold2 simulations demonstrated that the RBD-dimer arranged into an axisymmetric-like structure with its external structural domains extensively exposed (**Figure 2B**). Ferritin proteins spontaneously formed octahedral spherical particles (**Figure 2C**). To construct RBD-Fe nanoparticle vaccine, ST-RBD was incubated with equal mole of SC-Fe (**Figure 2D**).

To identify the nanoparticles, purified ST-RBD, SC-Fe, and assembled RBD-Fe were subjected to Western blotting using mouse anti-His antibodies and approximately 27 kDa ST-RBD dimer protein, 35 kDa SC-Fe protein, and 62 kDa RBD-Fe protein were observed. The results confirmed that RBD-Fe assembled successfully (**Figure 2E**). Moreover, SC-Fe and the assembled RBD-Fe nanoparticles were negatively stained, and TEM showed that SC-Fe and RBD-Fe could form spherical nanoparticles (**Figure 2F**). To further confirm the formation of nanoparticles, the colocalization of Alexa Fluor 488-labeled ST-RBD proteins and Alexa Fluor 594-labeled SC-Fe proteins were determined by confocal microscopy. The results showed that SC-Fe and RBD-Fe nanoparticles overlapped with orange-yellow fluorescence (**Figure 2G**). These results suggest that the nanoparticles were assembled through the SpyTag/SpyCatcher presentation strategy.

### 3.3 Protective antibodies induced by RBD-Fe nanoparticle in mice

**Figure 3:**
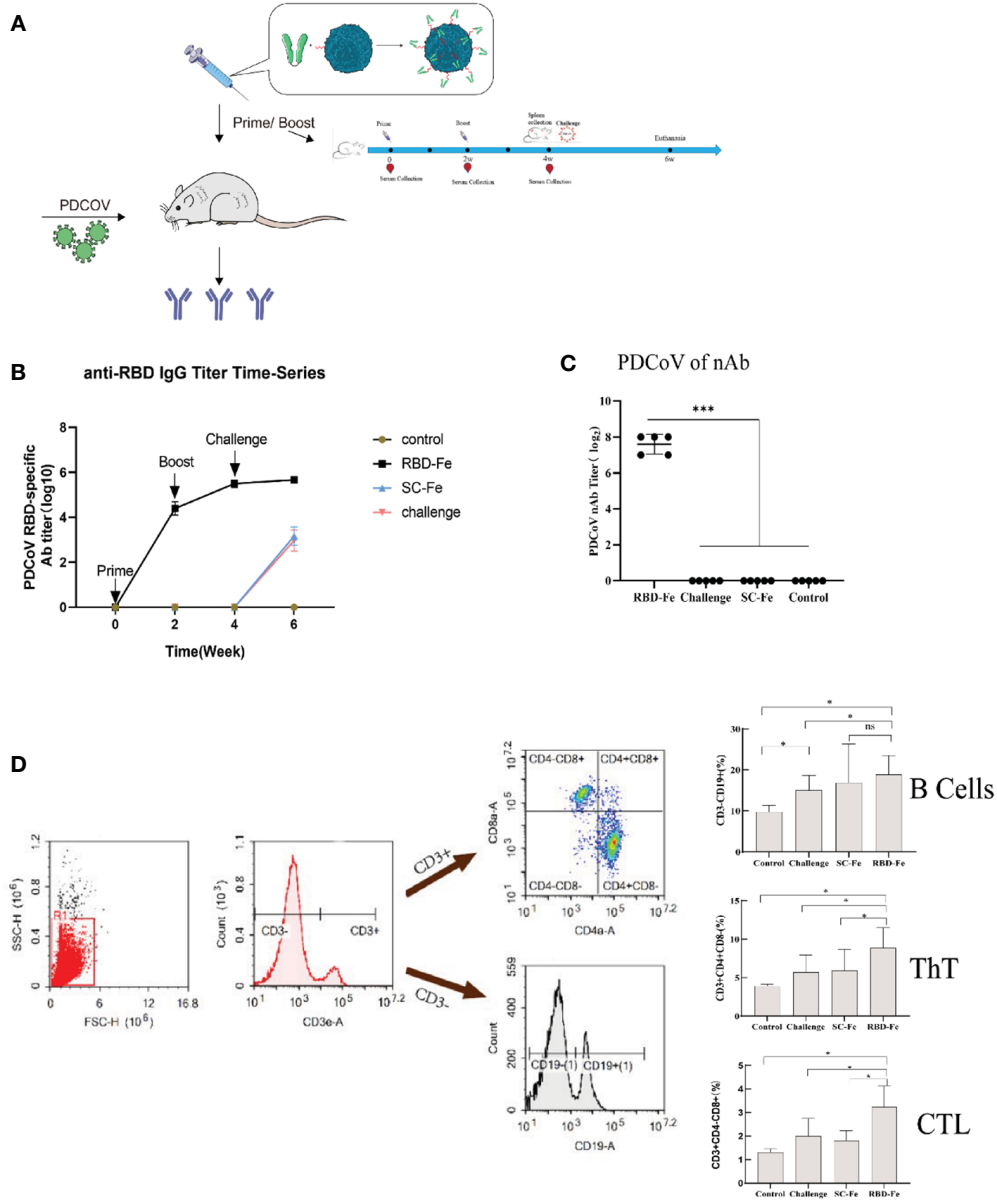
Immunogenicity of RBD-Fe nanoparticles. **(A)** Schematic flow diagram of the animal immunization procedure. Mice from each group were prime and boost-vaccinated at week 0 and week 2. Serum was collected every two week. The spleens were collected at week 6. Mice in SC-Fe, challenge and RBD-Fe groups were challenged orally with PDCoV at week 4 while control group treated with physical saline. **(B)** PDCoV-specific RBD-IgG titers of immunized mice at 0, 2, 4 and 6 week were detected by ELISA, and the IgG titers in each week were calculated and plotted as time-course curve. Bars represent the mean (standard deviation) of three replicates per treatment in one experiment. **(C)** The levels of neutralizing antibodies. The levels of neutralizing antibodies in mice serum at week 4 were determined using PDCoV strain JS2011 with a virus neutralization test. Bars represent the mean (standard deviation) of three replicates per treatment in one experiment. Statistical significance was indicated by *P < 0.05 (significant) compared with Control group. **(D)** Flow cytometry of splenocytes. At week 6, spleen cells were immunolabeled with antibody-fluorophore coupled antibodies to CD3e-PE, CD4-FITC, CD8a-PE-Cyanine7 and CD19-eFluorTM 450. B cells defined CD3^-^CD19^+^, ThT cells (CD3^+^CD4^+^CD8^-^) and CTL cells (CD3^+^CD4^-^CD8^+^). The numbers in the gates refer to the percentage of positive cells for each marker. Statistical significance was assessed using a one-way ANOVA followed by a Dunnett’s test (ns, non significance; *, *p* < 0.05; **, *p* < 0.01; ***, *p* < 0.001).

Since the RBD-Fe nanoparticles were constructed, mice were subcutaneously immunized to assess the immunogenicity of the nanoparticles (**Figure 3A**). First, the RBD-specific IgG antibody titers reach over 1:10^6^ at week 4, significantly higher than the other groups (**Figure 3B**). Furthermore, serum NA assay was performed and revealed that the neutralizing capability against PDCoV induced by the RBD-Fe nanoparticle vaccine was also dramatically increased at week 4 with a NA titer between 1:2^7^-1:2^8^, whereas the NA titers in the other groups remained below 1:2 (**Figure 3C**).

In order to address the adaptive immune responses in the immunized mice, immune cell populations were analyzed from splenocytes of the mice by flow cytometry. The results showed that the RBD-Fe group exhibited an increase in CD3^+^CD4^+^CD8^-^ and CD3^+^CD4^-^CD8^+^ T cell populations in the spleen compared to the SC-Fe groups on week 6. Also, CD3^-^CD19^+^ B cell populations increased in the RBD-Fe group compared to the other groups, suggesting that the RBD-Fe group generated a specific humoral immune response (**Figure 3D**).

### 3.4 RBD-Fe nanoparticle provides significant protection against PDCoV JS2211 challenge in mice

Necropsy was performed at week 6. In the RBD-Fe group, only one mouse (1/10) showed mild intestinal distension, while other organ examinations were normal. The Control group remained normal. In contrast, mice in the SC-Fe and Challenge groups displayed typical symptoms such as intestinal wall thinning, mesenteric and intestinal bleeding, and intestinal distension.

**Figure 4:**
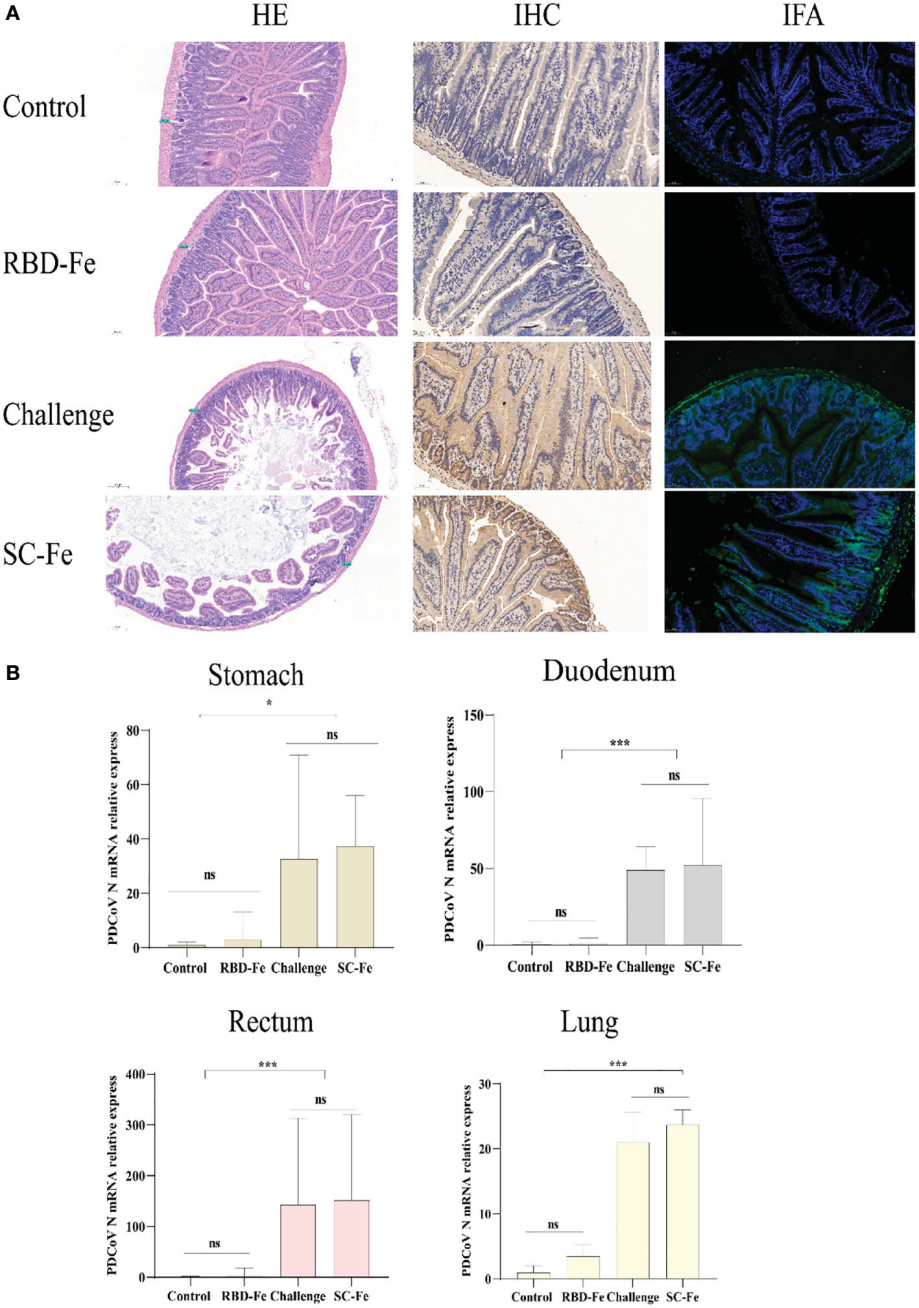
Histology and Viral RNA changes post‐challenge. **(A)** HE, IHC and IFA staining. HE results showed that SC-Fe and challenge mice exhibited mild villus atrophy in the duodenum post‐challenge. No obvious lesions were observed in the control and RBD-Fe mice post‐challenge. IHC and IFA analysis showed viral antigens were detected in the SC-Fe and challenge mice, no PDCoV N antigens were detected in the control and RBD-Fe group mice. **(B)** Detection of PDCoV N mRNA with RT‐qPCR in stomach, duodenum, rectum and lung. Statistical significance was assessed using a one-way ANOVA followed by a Dunnett’s test (ns, non significance; *, *p* < 0.05; **, *p* < 0.01; ***, *p* < 0.001).

Histopathological observations showed that the Control and RBD-Fe group exhibited a high density of villi, organized villi arrangement, thicker intestinal walls, and deeper crypts. In contrast, the Challenge group and SC-Fe group exhibited villus atrophy, fusion, shallow villus contraction, vacuolar degeneration, necrosis, and sloughing of villous tip epithelial cells, and degenerated intestinal epithelial cells detached into the lumen. Infiltration of a few lymphocytes and neutrophils in the lamina propria was observed, and the small intestine walls became thinner, and crypts shortened or disappeared (**Figure 4A**). Immunohistochemistry (IHC) and IFA using rabbit anti-PDCoV-N polyclonal antibodies revealed that, compared to the Control group, the RBD-Fe group exhibited minimal PDCoV antigen detection. In contrast, the SC-Fe group and Challenge group showed abundant PDCoV viral N protein, primarily localized to epithelial cells, consistent with previous research findings (30, 31). To further determine the distribution of the virus in different tissues of mice following PDCoV infection, qRT-PCR was used to assess the PDCoV mRNA in the lungs, stomach, duodenum, and rectum of mice. The results revealed that the RBD-Fe group exhibited significantly lower relative PDCoV mRNA in the lungs, stomach, rectum, and duodenum compared to the Challenge and SC-Fe groups (**Figure 4B**).

## 4 Discussion

It is reported that frequent recombination between different lineages may result in the emergence of PDCoV strains with divergent pathogenicity and host tropism (1, 32). PDCoV as a novel coronavirus has been reported to infect humans, suggesting that PDCoV has potential to proceed interspecies transmission or evolute as a new human strain (10). In this study, we isolated a newly PDCoV strain, JS2211. In order to analyze the genetic evolution of the PDCoV, we constructed a ML tree and found that the JS2211 strain was in latest China lineage. Kong et al. demonstrated that multiple PDCoV lineages, including US lineage, early Chinese lineage, Chinese lineage, and Vietnam/Laos/Thailand lineage, coexist in mainland China (33). Compared with the early Chinese lineage, the PDCoV JS2211 was closely related to the USA lineage, suggesting that the newly emerged PDCoV was spreading rapidly in the worldwide. Therefore, it is urgent to develop an effective vaccine to control the spread of PDCoV.

The efficacy of inactivated vaccine and subunit vaccine-based S protein or RBD have been investigated in several studies (34–36). Zhang et al. evaluated the protective efficacy of inactivated PDCoV vaccines in pregnant sows, with results showing an 87.1% protection rate in piglets (34). And the inactive PDCoV vaccine showed good immune effects in mice after injected in a third-boost manner (37). However, aluminum adjuvants commonly used in inactivated vaccines can lead to animal inflammation (31). Subunit vaccines based on synthetic peptides or recombinant proteins have the characteristics of high safety and strong immune targeting, and are currently a very promising vaccine in the world. In this study, we chose an E. coli expression system to express RBD protein, which can induce efficient immune response, as this is a commercially feasible system and can manufacture the candidate vaccine. Full-length trimeric S proteins often exhibit high immunogenicity, as they not only contain the receptor-binding domain (RBD) which is the primary target of neutralizing antibodies, but also non-RBD regions (residues 50-286 and 278-616 in the S1 subunit and residues 601-1087 in the S2 subunit) that can induce protective antibodies (30, 31, 38). However, it has been reported that various coronaviruses exhibit antibody-dependent enhancement (ADE) (39–41). Hence, seeking the minimal effective immunogen is a crucial strategy to enhance vaccine safety. But the small molecular size mono-RBD may face serious challenges in vaccine development, primarily due to their relatively low immunogenicity. Wang et al. evaluated the immunogenic efficacy of RBD region expressed by a baculovirus combined with different adjuvants through a prime-boost-second immunization regimen in mice (36). The results indicated that mice only exhibited an effective specific immune response after the third immunization, suggesting that mono-RBD has low immunogenicity, even though combined with adjuvants. Therefore, RBD should be optimized with appropriate adjuvants or multimerization modification. In this study, we employed a multimerization modification approach, creating an RBD-dimer that increases the antigen’s molecular weight to reach 27 kDa, which might make contribution to enhance immunoprotection in mice.

It was known that structure-guided antigen design is a key tool for rapid and precise subunit vaccine development (42, 43). Non-hemoglobin ferritin nanoparticles from Helicobacter pylori (H. pylori) have been successfully applied in influenza and COVID-19 nanoparticle vaccines, eliciting broad-spectrum neutralizing antibodies (26, 44). A previous study targeting HBV indicated that covalently coupled ferritin nanoparticles had higher expression levels than fusion expression (45). Furthermore, H. pylori ferritin differs significantly from mammal ferritin, making it less likely to induce autoantibodies. Therefore, we selected H. pylori ferritin as the scaffold of the PDCoV nanoparticle vaccine. We introduced the SpyTag/SpyCatcher system derived from Streptococcus pyogenes to covalently couple the ferritin nanoparticles instead of direct fusion expression, which is easily synthesized in large-scale and pure form, and greatly increased immunogenicity.

A Kunming mouse challenge model has been developed previously to investigate the pathogenicity of PDCoV JS2211 (27), and were used in this study to evaluate the immunoprotective effect of nanoparticle vaccines (RBD-Fe). The RBD-Fe elicited high level IgG and NA antibody titers (NA titer between 1:27-1:28, **Figure 3C**), which may play an important role in the protection against virulent PDCoV challenge in mice. In addition, T cell responses are also important for virus clearance, decreasing severe illness, and prognostic recovery (35, 46, 47). In this study, the nanoparticle vaccine (RBD-Fe) elicited not only high-level antibodies titers but also high percentage of CD3+CD4+CD8- and CD3+CD4-CD8+ T lymphocytes in Kunming mice (**Figure 3D**). The strong T cell immune responses activated by RBD-Fe may help to eliminate the infected cells, thereby contributing to the protection for mice against PDCoV. The challenge assay showed that the mice in the RBD-Fe group were detected the lower viral RNA copies in the lung, stomach, and intestinal tissues compared with those in other groups. Moreover, only one mouse in RBD-Fe group displayed mild pathological changes of intestinal tissues, whereas all mice in other control groups displayed serious pathological damage of intestinal tissues. Li et al.’s study showed that S-based subunit vaccine induced high level NAbs (about between 1:2^6^-1:2^8^) and cellular immune responses post two immunizations in mice, similar to that did by RBD-Fe in this study, and provided significant protection for newborn piglets in the following passive immunity (35). These results suggested the nanoparticle vaccine (RBD-Fe) may provide significant protection for newborn piglets in the passive immunity experiment, which will be performed in the future.

In conclusion, we constructed RBD-Fe nanoparticles, which could induce high level cellular and humoral immune responses, and provide almost full protection against virulent PDCoV challenge in mice. Therefore, RBD-Fe nanoparticle is a promising vaccine candidate against PDCoV.

## Data availability statement

The datasets presented in this study can be found in online repositories. The names of the repository/repositories and accession number(s) can be found below: https://www.ncbi.nlm.nih.gov/genbank/, MW349841.1.

## Ethics statement

The animal study was approved by the Experimental Animal Care and Use Committee of the Shanghai Veterinary Research Institute, Chinese Academy of Agricultural Sciences(No. SV-20230512-02). The study was conducted in accordance with the local legislation and institutional requirements.

## Author contributions

YW: Writing – original draft. JS: Methodology, Writing – original draft. XD: Methodology, Writing – original draft. JW: Methodology, Writing – original draft. MZ: Software, Writing – original draft. LuY: Data curation, Writing – original draft. PT: Formal Analysis, Writing – original draft. HL: Investigation, Writing – original draft. YZ: Resources, Writing – original draft. GT: Funding acquisition, Writing – original draft. GL: Writing – review & editing. LxY: Writing – review & editing.
