# Supplementary material for: Nanoparticle vaccines based on the receptor binding domain of porcine deltacoronavirus elicit robust protective immune responses in mice
Source: Front Immunol. 2024 Mar 14;15:1328266. doi: 10.3389/fimmu.2024.1328266 (PMC10972852; doi:10.3389/fimmu.2024.1328266)
Supplement: Supplementary file 1 [file Table_1.docx]

**The nucleic acid sequence of ST-RBD:**

***GCGCATATTGTGATGGTGGATGCGTATAAACCGACCAAA*TTTGCAACCGCAGTACAGGCACGTCTGAATTATGTTGCACTGCAGACCAATGTTCTGCAGGAAAATCAGAAAATTCTGGCAGAAAGCTTTAATCAGGCAGTTGGTAACATTAGCCTGGCGCTGAGCAGTGTTAATGATGCTATCCAGCAGACCAGCGAAGCACTGAATACCGTTGCAATTGCAATTAAAAAGATCCAGACAGTTGTTAATCAGCAGGGCGAAGCACTGAGCCATCTGACCGCACAGCTGAGTAATAATTTTCAGGCAATCTCAACCAGCATTCAGGATATTTATAACCGTCTGGAA**GGTGGCGGTGGCAGTGGTGGTGGTGGTAGT**TTTGCAACCGCGGTTCAGGCACGTCTGAACTATGTTGCACTGCAGACAAACGTTCTGCAGGAAAACCAGAAAATTCTGGCTGAAAGTTTTAATCAGGCGGTTGGTAATATTAGTCTGGCGCTGTCTAGTGTGAATGATGCAATTCAGCAGACCTCTGAAGCACTGAACACCGTTGCAATCGCAATTAAAAAGATTCAGACAGTTGTGAATCAGCAGGGTGAAGCACTGTCACATCTGACCGCCCAGCTGAGCAATAACTTTCAGGCAATTTCTACCAGCATTCAGGATATCTATAACCGTCTGGAA**

Notes:The italicized words are the **sequence of spytag;**The highlighted words in yellow are the **sequence of RBD;**The highlighted words in red are the **sequence of linker.**
